# Association between visceral adiposity index and infertility in reproductive-aged women in the United States

**DOI:** 10.1038/s41598-024-64849-0

**Published:** 2024-06-20

**Authors:** Jiaru Zhuang, Yuan Wang, Shan Wang, Renjing Hu, Yibo Wu

**Affiliations:** 1 Human Reproductive Medicine Center, Affiliated Hospital of Jiangnan University, 1000 Hefeng Road, Wuxi, 214026 Jiangsu People’s Republic of China; 2 Department of Laboratory Medicine, Jiangnan University Medical Center, 68 Zhongshan Road, Wuxi, 214000 Jiangsu People’s Republic of China

**Keywords:** Infertility, Visceral adiposity index, Obesity, Non-linear, NHANES, Diseases, Reproductive disorders, Infertility

## Abstract

Over the years, obesity has become more commonplace and has had a substantial impact on several medical specialties, including reproductive medicine. The potential correlation between the visceral adiposity index (VAI) and infertility has yet to be determined. Women between the ages of 18 and 45 were included in this cross-sectional study, which was conducted as part of the National Health and Nutrition Examination Survey (NHANES) between 2015 and 2020. Three tertiles were used to group VAI levels. Subgroup analysis and weighted binary logistic regression were employed to investigate the independent relationship between VAI and infertility. Smooth curve fitting was used to explore nonlinear relationships. This cross-sectional study followed the criteria of the STROBE guidelines. Of the 1231 participants, 127 were infertile women aged 18–45 years. A higher VAI was associated with a higher prevalence of infertility (OR = 1.22, 95% CI:1.03–1.45), which remained consistent across all subgroups (p > 0.05 for all interactions). We demonstrated a positive nonlinear association between VAI and infertility using a smooth curve fit. A higher visceral adiposity index level is positively correlated with a higher incidence of infertility among women in the United States. Women who are infertile can be identified using the visceral obesity index, and controlling visceral obesity may help lower the chances of becoming infertile.

## Introduction

Infertility is a medical condition usually defined as the failure to conceive after 12 months of regular sexual intercourse^1^. About 7 to 15.5% of women in the US who are of reproductive age have infertility, and 8 out of 12 couples struggle with conception^2^. Infertility affects a sizable percentage of people worldwide (9.0%), in rich countries (3.5–16.6%), and in developing countries (6.9–9.3%)^3^. Even though infertility has gained international attention recently as a public health concern, the factors that contribute to it still need to be further investigated.

One prevalent issue among women who are fertile is obesity. It is generally regarded as an excessive build-up of body fat that has a detrimental impact on one’s health^4,5^. It is predicted that by 2025, more than 21% of women worldwide will be obese. This tendency may be linked to both rapid changes in lifestyle and economic development^6^. More gynecological disorders in women, such as excessive menstruation^7^, endometriosis and uterine fibroids (UF)^8,9^, polycystic ovary syndrome (PCOS)^10,11^, pregnancy complications like pre-eclampsia and eclampsia^12^, miscarriage^13^ and infertility^14,15^, are linked to higher body mass indices (BMI). Despite being a conventional and cost-effective approach, using body mass index (BMI) to evaluate obesity (defined as a BMI exceeding 25 kg/m^2^) lacks the ability to differentiate between lean and fat body mass^16,17^. Because BMI does not provide a good picture of obesity distribution, it is not appropriate to use it alone to assess obesity^18^. Waist circumference measures central obesity, but it may not adequately reflect the health hazards associated with abdominal obesity since it cannot distinguish between visceral and subcutaneous adipose tissue in the abdomen. A recently proposed mathematical model with a scientific design, the visceral obesity index (VAI) evaluates the quantity and function of visceral fat in a person’s body^19^. Increased visceral fat has been linked in studies to a number of diseases, including metabolic syndrome, diabetes, cardiovascular disease, and several forms of cancer^20^. Anthropometric information (waist circumference, BMI) and metabolic markers (triglycerides and HDL cholesterol) are combined in the VAI assessment approach. In essence, VAI offers a comprehensive evaluation of a person's visceral fat status and could be a helpful substitute for visceral CT scans due to its lower radiation risks and cost^21^.

This study investigated the connection between infertility and the visceral obesity index in American women between the ages of 18 and 45. The National Health and Nutrition Examination Survey (NHANES) will provide the data for the study. The goal is to shed light on the complex relationship between visceral fat and infertility in order to aid in the clinical development of treatments aimed at reducing the risk of infertility.

## Materials and methods

### Data source

The National Center for Health Statistics (NCHS) is the publishing organization for the National Health and Nutrition Examination Survey (NHANES), a national survey that evaluates Americans’ health and nutrition. To ensure that the sample was representative, a complicated, multistage probability design was used to perform the NHANES^22^. To gather information on the participants’ socioeconomic situation, health, and other aspects, a household interview was conducted. Both laboratory and physical examinations were conducted in a mobile examination center. The NCHS study Ethics Review Board authorized all NHANES study procedures, and all survey participants gave written informed consent. The public can access all the information regarding the NHANES study design and data at www.cdc.gov/nchs/nhanes/. This cross-sectional study followed the STROBE reporting standards^23^.

### Study population

**Figure 1:**
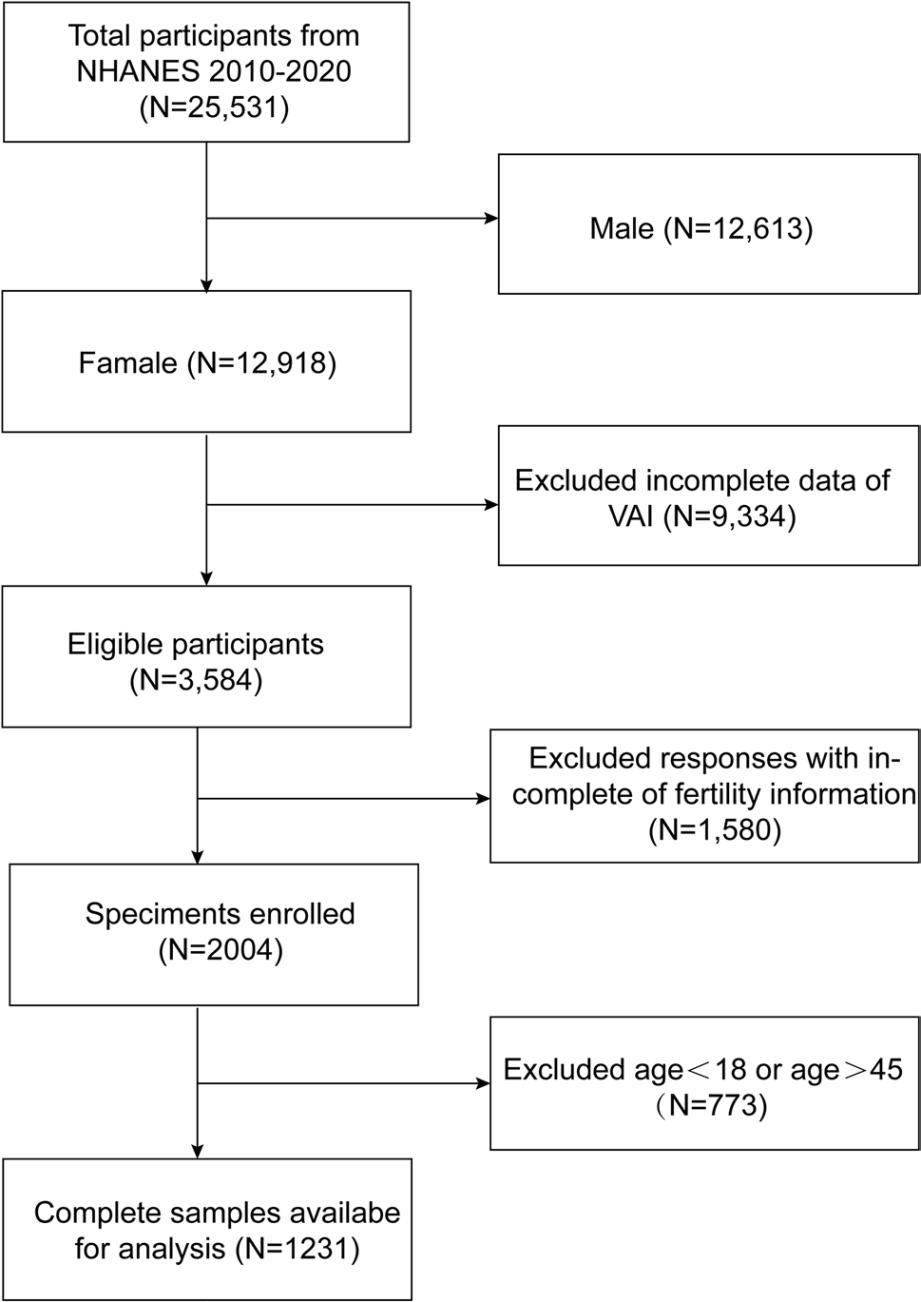
Flow chart of the inclusion and exclusion of study participants.

Data from the National Health and Nutrition Examination Survey (NHANES) covering the years 2015–2020 was utilized. We included in our research participants who provided comprehensive information on their visceral obesity index (VAI) and infertility. A total of 25,531 people were initially enrolled. After excluding male participants (n = 12,613), individuals without information on waist circumference (n = 143), triglycerides (n = 9141), BMI (n = 50), individuals without information on infertility (n = 1580), and female participants over 45 and under 18 (n = 773), our final analysis comprised 1231 eligible participants (Fig. 1).

### Calculation of VAI

Amato et al. developed the gender-specific visceral obesity index (VAI) to account for physiological variations in visceral obesity between men and women^24^. VAI is a measure of anthropometric and metabolic characteristics, such as high-density lipoprotein cholesterol (HDL-C), triglycerides (TG), waist circumference (WC), and body mass index (BMI). It is thought to be a sign of malfunction and buildup of visceral adipose tissue.

The VAI for each participant was calculated by using the following formulas. For males: VAI = WC/(39.68 + (1.88*BMI))*(TG/1.03)*(1.31/HDL-C); For females: VAI = WC/(36.58 + (1.89*BMI))*(TG/0.81)*(1.52/HDL-C). TG and HDL-C were calculated in mmol/L, and WC was calculated in cm in the formulas.

### Assessment of infertility

The dependent variable for infertility was each woman’s self-report from the Reproductive Health Questionnaire (questionnaire variable name: RHQ074). Researchers prodded participants with questions like, “Have you tried to get pregnant for one year?”^25^. A “infertile” situation was indicated if the response was “yes,” and a “fertile” situation was indicated if it was no.

### Covariates

Based on similar literature and practice^26–28^, factors included age, ethnicity, education level, marital status, poverty income ratio (PIR), diabetes, hypertension, alcohol and smoking patterns, waist circumference, BMI, triglycerides, and HDL cholesterol. There were five racial and ethnic groupings among the participants. Mexican Americans, Blacks, Whites, non-Hispanics, Hispanics, and Others (including Multiracial). Waist circumference, body mass index, triglycerides, HDL cholesterol, age in years, and poverty income ratio (PIR) were among the continuous variables that were measured. The definitions of the three degrees of education were as follows: high school graduate/GED or equivalent, above high school, and below high school^29^. The NHANES maintained its classification of marital status into five categories: cohabiting, single, widowed, divorced, married, and unmarried. Diabetes was described as a diagnosed illness^30^. Additionally, the measure of hypertension was self-reported^31^. Those who never smoked (less than 100 cigarettes in their lifetime), those who smoked in the past (at least 100 cigarettes in their lifetime, smokers, and no smokers at all), and those who now smoke (more than 100 cigarettes in their lifetime or smoked daily) were the three categories depending on their smoking status. A person's drinking status was ascertained by asking if they had more than 12 drinks in a year. Those who answered "yes" were deemed to be drinkers, while those who answered “no” were not.

### Statistical analysis

All statistical analyses were performed with consideration for the intricate, multistage clustered surveys and with the appropriate NHANES sampling weights, following the recommendations of the Centers for Disease Control and Prevention.

In descriptive analyses, a weighted Student’s *t*-test (for continuous variables) or a weighted Chi-square test (for categorical data) was used to evaluate the two comparison groups based on their infertility status. For continuous data, proportions were utilized to represent categorical parameters, while averages and standard deviations were employed to describe them. Multivariate regression models using the NHANES complex sample design (sampling weights) were used to examine the link between VAI and infertility. In Model 1, covariates were left unchanged. Model 2 adjusted for age and race. Model 3 took into consideration the following variables: age, race, education level, marital status, number of cigarettes smoked in the past 100 days, number of beverages consumed annually, diabetes (yes/no), and hypertension (yes/no). We used smoothed curve fitting in addition to subgroup analyses to look at the nonlinear relationship between VAI and infertility.

We used R (http://www.r-project.org) and Empower software (http://www.empowerstats.com) for all statistical analyses, following the Centers for Disease Control and Prevention’s (CDC) instructions. The statistical significance criterion was established at *p* < 0.05.

## Results

### Baseline characteristics of study participants

Of the 1,231 participants, 127 were infertile women between the ages of 18 and 45, and 116 of the infertile women had regular menstruation over a 12 month period. The range of VAI for tertiles 1–3 were 0.1–0.65(≤ 0.65),0.65–1.75(≤ 1.75),and 1.75–19.69(≤ 19.69).Table 1 displays the characteristics of the study participants, categorized based on whether they were infertile or not. Women who were older, married or living together, drank alcohol, had a higher body mass index, and had a larger waist circumference were more likely to self-report being infertile. Furthermore, women with a higher VAI also had a higher prevalence of self-reported infertility, with a mean of 1.72 ± 1.91.

**Table 1:** Baseline characteristics of study participants (N = 1231).

	Infertility	Control	p-value
	N = 127	1104	
Age, mean ± SD (years)	33.33 ± 6.89	30.62 ± 7.84	< 0.001
poverty income ratio (PIR)	2.37 ± 1.58	2.27 ± 1.58	0.412
Race [n (%)]			
Mexican American	22 (17.32%)	172 (15.58%)	0.813
Other Hispanic	10 (7.87%)	118 (10.69%)	
Non-Hispanic White	40 (31.50%)	317 (28.71%)	
Non-Hispanic Black	30 (23.62%)	283 (25.63%)	
Other race—including multi-racial	25 (19.69%)	214 (19.38%)	
Education level [n (%)]			0.887
Below highschool	18 (14.52%)	146 (14.61%)	
Highschool grad/GED/equivalent	22 (17.74%)	195 (19.52%)	
Above highschool	84 (67.74%)	658 (65.87%)	
Marital status [n (%)]			< 0.001
Married	90 (72.58%)	490 (49.05%)	
Widowed	9 (7.26%)	56 (5.61%)	
Divorced	13 (10.48%)	264 (26.43%)	
Separated	0 (0.00%)	11 (1.10%)	
Never married	7 (5.65%)	125 (12.51%)	
Living with partner	5 (4.03%)	53 (5.31%)	
Smoking status [n (%)]			0.926
Now	20 (42.55%)	126 (44.37%)	
Former	6 (12.77%)	31 (10.92%)	
Never	21 (44.68%)	127 (44.72%)	
Alcohol drinking [n (%)]			0.008
Yes	15 (13.51%)	60 (6.59%)	
No	96 (86.49%)	851 (93.41%)	
Hypertension [n (%)]			0.001
Yes	29 (22.83%)	137 (12.41%)	
No	98 (77.17%)	967 (87.59%)	
Diabetes [n (%)]			0.001
Yes	15 (11.81%)	39 (3.53%)	
No	112 (88.19%)	1065 (96.47%)	
TG (mmol/L)	1.02 ± 0.73	0.94 ± 0.62	0.016
HDL (mmol/L)	1.35 ± 0.38	1.47 ± 0.40	< 0.001
BMI (kg/m^2^)	32.69 ± 8.60	29.42 ± 8.53	< 0.001
WC (cm)	103.61 ± 20.52	94.92 ± 19.12	< 0.001
VAI	1.72 ± 1.91	1.44 ± 1.30	< 0.001

Mean ± SD for continuous variables: p value was calculated by the weighted linear regression model.

% for Categorical variables: p value was calculated by the weighted chi-square test.

### Relationship between visceral obesity index and infertility

**Figure 2:**
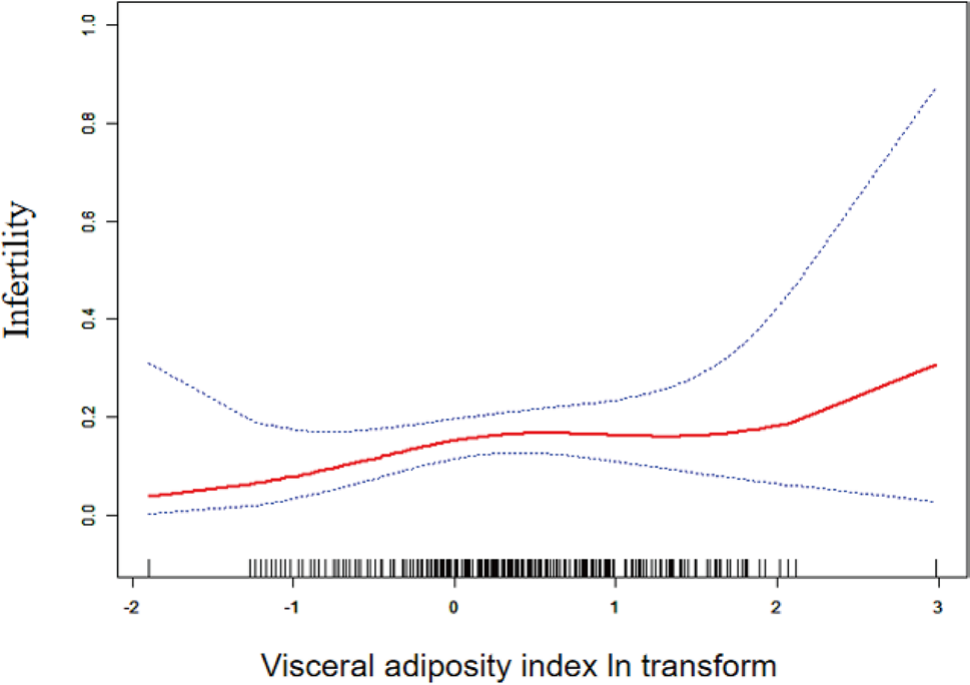
Smoothing curve fitting of AVI index and infertility.

The correlation between VAI and infertility is shown in Table 2. Our results imply that a higher risk of infertility is linked to a higher VAI. There was a positive correlation between VAI and infertility in Models 1, 2, and 3. According to the fully adjusted model (Model 3: OR = 1.22, 95% CI:1.03–1.45), there was a 22% increase in the probability of being infertile for every unit increase in VAI. The statistical significance of this link persisted even after dividing VAI into thirds. Those in the highest VAI tertile were at a 252% higher risk than those in the lowest VAI tertile (OR = 3.52, 95% CI:1.18–10.49; *p* = 0.02) (Table 2). Additionally, we used smoothed curve fitting to further examine the relationship between VAI and the risk of infertility, and the results indicated a positive nonlinear relationship (Fig. 2).

**Table 2:** Associations between weight-adjusted-waist index and the risk of infertility.

		OR (95% CI), P-value		
		Crude model	Minimally adjusted model	Fully adjusted model
		(Model 1)^1^	(Model 2)^2^	(Model 3)^3^
N (infertility)				
Continuous	127	1.12 (1.00, 1.25) 0.04	1.12 (1.00, 1.25) 0.04	1.22 (1.03, 1.45) 0.02
Categories				
Tertile1	23	Reference	Reference	Reference
Tertile2	46	2.10 (1.25, 3.53) < 0.01	2.00 (1.19, 3.39) < 0.01	2.72 (0.90, 8.26) 0.07
Tertile3	58	2.71 (1.64, 4.49) < 0.01	2.41 (1.43, 4.06) < 0.01	3.52 (1.18, 10.49) 0.02

Insensitivity analysis, the visceral adiposity index was converted from a continuous variable to a categorical variable (tertiles).

*95% CI* 95% confidence interval. *OR* odds ratio.

^1^Model 1: Covariates were not adjusted at all.

^2^Model 2: Adjusted forage, and race.

^3^Model 3: Adjusted forage, ratio of family income to poverty, race, education level, marital status, Smoking status, Alcohol drinking, Hypertension, Diabetes.

### Subgroup analysis

We performed subgroup analyses to determine whether other factors altered the association between VAI and infertility. The results indicated that there was no dependence and that the subgroup between VAI and infertility was stable. The positive correlation between VAI and infertility was not significantly impacted by stratified factors such as age, ethnicity, education level, marital status, smoking at least 100 cigarettes, drinking alcohol at least 12 times a year, diabetes, and hypertension (*p* > 0.05), as shown in Table 3.

**Table 3:** Subgroups analyses of the effect of VAI on infertility.

Subgroups	N		OR (95% CI)	P for interaction
	Total	Infertility		
Age				0.2049
Tertile1	408	21	1.28 (0.68, 2.43)	
Tertile2	378	43	1.86 (1.06, 3.26)	
Tertile3	445	63	1.28 (1.03, 1.60)	
Race				0.3855
Mexican American	194	22	1.15 (0.71, 1.86)	
Other Hispanic	128	10	1.69 (0.88, 3.24)	
Non-Hispanic White	357	40	1.27 (1.01, 1.59)	
Non-Hispanic Black	313	30	1.29 (0.74, 2.25)	
Other race	239	25	0.64 (0.26, 1.57)	
Education level				0.2534
Below high school	164	18	1.63 (0.93, 2.85)	
High school grad/GED/equivalent	217	22	1.00 (0.56, 1.77)	
Above high school	742	84	1.12 (0.82, 1.51)	
Marital status				0.5472
Married	580	90	1.20 (0.90, 1.60)	
Widowed	65	9	1.37 (0.99, 1.90)	
Divorced and separated	288	13	0.67 (0.26, 1.71)	
Never married	132	7	1.06 (0.49, 2.31)	
Living with partner	58	5	1.08 (0.33, 3.54)	
Smoking status				0.5515
Now	146	20	1.09 (0.66, 1.81)	
Former	37	6	1.34 (0.72, 2.48)	
Never	148	21	1.10 (0.82, 1.47)	
Alcohol drinking				0.8586
Yes	75	15	1.22 (1.02, 1.45)	
No	947	96	1.15 (0.59, 2.22)	
Hypertension				0.2074
Yes	166	29	1.26 (0.86, 1.83)	
No	1065	98	0.97 (0.73, 1.29)	
Diabetes				0.8003
Yes	54	15	1.16 (0.85, 1.59)	
No	1177	112	1.10 (0.81, 1.48)	

Subgroup analysis of the correlation between visceral obesity index and infertility. Factors such as age, race, education level, marital status, smoking, alcohol consumption, diabetes, and hypertension did not affect the positive correlation between visceral obesity index and infertility.

## Discussion

We recruited 1231 female patients for this cross-sectional study, and the results showed a positive correlation between VAI and infertility, independent of age, race, education level, marital status, smoking, alcohol intake, diabetes, or hypertension. According to the findings of the investigation, lowering VAI levels may help decrease the likelihood of infertility.

This is the only study that we are aware of that assesses the correlation between VAI and female infertility. Obese women often experience irregular menstruation with poor ovulation, endometriosis, and infertility, which highlights the detrimental consequences of obesity on reproduction^32,33^. Numerous studies have demonstrated that obesity not only deteriorates metabolic status but also causes ovulatory dysfunction, increasing the incidence of infertility in obese women by three times compared to non-obese women^34^. Especially two investigations including sizable cohorts of Danish women who were considering becoming pregnant revealed an unfavorable relationship between higher BMI and fertility^25^. Notably, obese women still have low fertility even in the absence of ovulatory dysfunction. Gesink and colleagues examined a large American cohort of over 7000 women and discovered that the probability of spontaneous conception declined linearly with BMI > 29 kg/m^2^. Similar findings were found in a comparable study that involved over 3000 women in the Netherlands who had regular menstrual cycles^35^. Furthermore, it seems that participation in assisted conception programs reduces the fertility of obese women^36^. In fact, poor oocyte quality and reduced preimplantation have been linked to poor results in individuals undergoing in vitro fertilization (IVF) who are overweight or obese^37^. Losing weight is therefore highly advised in these women in order to enhance reproductive function^38^. Our latest research supports and validates the detrimental effect of visceral fat on infertility in women.

There is a connection between declining metabolism and visceral adiposity. In Yu Kang et al.’s study, patients who were metabolically unwell and obese (MUO) had a considerably greater VAI than patients who were healthy and obese (MHO). Furthermore, VAI and the frequency of conversion to the MUO phenotype correlated positively^39^. Insulin sensitivity did not correlate with waist circumference or BMI in a study by Amato et al., but there was a positive connection between VAI and cardiometabolic risk as well as visceral adipose tissue measured by magnetic resonance imaging^19^. An elevated VAI was linked to an increased cardiometabolic risk in a research involving 1764 hospitalized patients^24^. Studies have shown that TG/HDL-C is a strong predictor of metabolic syndrome and insulin resistance, playing a crucial role in the development of infertility^40^. The development of infertility and metabolic disorders is complex and diverse, and lipid metabolism disorders may play a key role in follicular development, egg maturation, and hormone secretion^41^. A large number of animal studies have confirmed that dyslipidemia can lead to a decrease in female reproductive capacity^42^. Therefore, VAI is able to indicate abnormal metabolic status in the body, thereby predicting infertility risk and serving as a basis for health promotion.

VAI has been shown to be a predictor of clinical severity and treatment outcome in patients with polycystic ovary syndrome^21^. The association between VAI and infertility is not well understood, but our findings suggest that increased VAI is associated with an increased risk of potential infertility, primarily because of neuroendocrine mechanisms that interfere with ovarian function and can affect ovulation rates and endometrial tolerance^33^. The circulating levels of gonadotropins, estradiol, and estradiol during the follicular phase are lower in obese women, even with normal menstrual cycles and apparently normal fertility. This suggests that the obesity condition itself has an inhibitory influence on the production of these hormones^43^. All systems involved in oocyte differentiation and maturation (including hormones, proteins, and soluble substances secreted by adipocytes) are dysregulated and impacted in their physiology because obesity is pathologically related to inflammation^44^. The reduction of women's fertility potential due to adipose tissue is therefore directly caused by malfunctioning of the primary molecular mechanisms that control the normal biological activity of the cellular components of their reproductive organs, which are also regulated by the hypothalamic-pituitary-ovarian axis^11^.

There are various benefits to our study. Initially, the NHANES database served as the foundation for our investigation, and every analysis considered the use of suitable NHANES sampling weights to increase the representativeness of the findings. Second, we investigated the nonlinear link between infertility and VAI by sensitivity analysis. This is the first study to look at the relationship between VAI and female infertility. Third, VAI is a quick and uncomplicated clinical tool that should be used to advise women of reproductive age about their higher risk of infertility at medical reviews. Our study is not without limits, though. First, we were unable to clearly determine a causal association among VAI and infertility because of the cross-sectional nature of our study. Secondly, an in-depth examination of additional markers was not feasible due to the restricted data present in the NHANES database.

## Conclusion

According to our research, higher VAI is associated with a higher incidence of infertility. Therefore, high VAI levels may be associated with an increased risk of infertility, and VAI provides a practical and easily accessible method to assess metabolic and reproductive problems in infertile women. However, more large-scale prospective studies are needed in the future to confirm the results of this study.

### Supplementary Information

Supplementary Information.

## Data Availability

All data generated or analysed during this study are included in Supplementary Material.
